# Prevalence of thrombocytopenia before and after initiation of HAART among HIV infected patients at black lion specialized hospital, Addis Ababa, Ethiopia: a cross sectional study

**DOI:** 10.1186/s12878-018-0103-6

**Published:** 2018-05-09

**Authors:** Gashaw Garedew Woldeamanuel, Diresibachew Haile Wondimu

**Affiliations:** 1 Department of Medicine, College of Medicine and Health Sciences, Wolkite University, Ethiopia, P.O. Box 07, Wolkite, Ethiopia; 2 Department of Medical Physiology, School of Medicine, College of Health Sciences, Addis Ababa University, Ethiopia, Addis Ababa, Ethiopia

**Keywords:** HIV, HAART, Thrombocytopenia, Ethiopia

## Abstract

**Background:**

Hematological abnormalities are common in HIV positive patients. Of these, thrombocytopenia is a known complication which has been associated with a variety of bleeding disorders. However, its magnitude and related factors have not been well-characterized in the era of highly active antiretroviral therapy (HAART) in Ethiopia. Therefore, this study aimed to assess the prevalence of thrombocytopenia before and after initiation of HAART among HIV positive patients attending Black Lion Specialized Hospital, Addis Ababa, Ethiopia.

**Methods:**

A cross sectional study was conducted from February to April 2017 in Black Lion Specialized Hospital, Addis Ababa, Ethiopia. A total of 176 patients on HAART were selected using simple random sampling techniques. Socio-demographic and clinical characteristics of the study patients were collected using structured questionnaire. Measurements of platelet counts and CD4 + T cell counts were made using Sysmex XT 2000i hematology analyzer and BD FACS Count CD4 analyzer, respectively. Statistical analysis of the data (Paired T- test and binary logistic regression) was done using SPSS version 20. *P*-value < 0.05 was considered as statistically significant.

**Results:**

A total of 176 patients (Age > 18 years old) were enrolled in this study and had a mean age of 40.08 ± 9.38 years. There was significant increase in the mean values of platelet counts (218.44 ± 106.6 × 10^3^/μl vs 273.65 ± 83.8 × 10^3^/μl, *p* < 0.001) after six months of HAART initiation compared to the baseline. Prevalence of thrombocytopenia before and after HAART initiation was 25 and 5.7% respectively. HIV patients whose CD4 counts < 200 Cells/μl were more likely to have thrombocytopenia than HIV patients whose CD4 count ≥350 Cells/μl. However, it was not statistically associated with prevalence of thrombocytopenia.

**Conclusions:**

This study has shown that the prevalence of thrombocytopenia after HAART initiation was decreased significantly. Based on our results, a number of study participants still had thrombocytopenia after initiation of HAART. Therefore, continuous screening for thrombocytopenia among HIV infected patients should be performed to decrease the risk of morbidity and mortality.

**Electronic supplementary material:**

The online version of this article (10.1186/s12878-018-0103-6) contains supplementary material, which is available to authorized users.

## Background

Thrombocytopenia is a frequent hematologic disorder in patients infected with the human immunodeficiency virus (HIV) [1]. It can occur independently of other cytopenias and at all stages of HIV infection [2]. Although often asymptomatic, the thrombocytopenia in these patients may be associated with a serious complication including major bleeding and death [3]**.** It is a frequent disorder occurring in about 30–40% of individuals with HIV infection [4]. Thrombocytopenia may indicate the initial manifestation of HIV diseases and it may enhance the progress of the disease into AIDS or advanced immunological deterioration [5].

The underlying mechanisms for the development of thrombocytopenia has not yet been well described [6]. The suggested mechanisms that may account for the development thrombocytopenia includes; increased destruction of platelets due to the presence of anti-platelet antibodies, and direct infection of megakaryocytes by HIV leading to low production of platelets from those precursor cells [6].

Multicenter AIDS cohort study was conducted in Asia, America and Africa [7]. The result showed that the average frequencies of thrombocytopenia at initiation of antiretroviral therapy were 7% and varied by country [7]. For instance, a study conducted in India showed that the prevalence of thrombocytopenia before initiation of Zidovudine was 16.6% which rises to 30% after initiation of Zidovidine. It was suggested mechanistically that immune mediated destruction of both platelets and megakaryocytes occurs in Zidovudine therapy [8].

The prevalence of thrombocytopenia showed an increasing trend with decreasing CD4 count [6, 9] but, the prevalence of thrombocytopenia did not differ by sex, ethnicity or age [9]. A study conducted in Uganda also reported that the prevalence of thrombocytopenia was 17.8% among HAART naive and was 13.0% for clients who were on ART for up to 6 months. The study found a significant association between thrombocytopenia and other cytopenias, CD4^+^ T cell counts, antiretroviral treatment(ART), and deteriorating HIV stage [10].

A comparative cross sectional study carried out at Gondar University hospital, Ethiopia showed that the prevalence of thrombocytopenia was 9% in HAART naïve patients and 4.1% in patients on HAART [11].

Although cytopenias have been widely reported in HIV infection, there is little data regarding prevalence and associated factors of thrombocytopenia among HIV infected patients before and after initiation of HAART in Ethiopia. This study will provide further information and it can serve as a reference material for further researches with regards to HIV related thrombocytopenia. The aim of this study was therefore to determine the prevalence of thrombocytopenia before and after initiation of antiretroviral therapy among HIV patients who attended at ART clinic of Black Lion Specialized Hospital, Addis Ababa, Ethiopia.

## Methods

The methodological approach of this study is summarized based on previous study [12]. Institution based cross sectional study design was conducted in Black Lion Specialized Hospital, Addis Ababa, Ethiopia from February to April 2017. During the data collection period, a total 2675 HIV infected adults were on ART, of which 176 HIV infected patients taking HAART for at least six months were selected randomly. Sample size was determined using a statistical formula for single population proportion (*n* = Z^2^ p(1-p) / d^2^), taking *p* = 12.7% (prevalence rate of thrombocytopenia from previous study) [4], 5% level of precision (d) with 95% confidence interval. Pregnant women, patients transferred from other health institutions, diagnosed as having hematological diseases, severely sick due to other medical conditions and those who took other medication were excluded from the study.

The structured questionnaire was adapted (see additional file 1) after the review of different literatures and the data was collected by trained ART nurses. Data concerning socio-demographic, clinical characteristics and pre ART information of the study participants were collected by interviewer administered questionnaire and review of medical records. Then, blood sample was collected and sent to the hematology laboratory. Based on the standard procedures, platelet counts and CD4+ T cell counts were determined using Sysmex XT 2000i hematology analyzer and BD FACS Count System respectively.

To maintain good quality of the data; standard procedures were followed during all laboratory procedures and the quality of CD4 and hematology analyzer were checked by running quality control samples along the patients sample.

Additionally, there was training of data collectors, pre testing of questionnaires and the data collection process were supervised in daily fashion.

Thrombocytopenia was defined as platelet counts less than 150,000cells/μl. It was further classified into mild (100,000–150,000/mm^3^), moderate (50,000/mm^3^–100,000/mm^3^) and severe thrombocytopenia (platelet counts < 50,000/mm^3^). The data were coded, checked and entered into SPSS version 20 for analysis. Descriptive statistics (mean and standard deviation) were used for continuous variables in the course of analysis. To assess the association between dependent variables and independent variables, logistic regression was done. A *p*- value of < 0.05 was considered to be statistically significant.

## Result

### General characteristics of study participants

A total of 176 HIV positive patients, of which 107(60.8%) women and 69(39.2%) men were involved in this study. The mean age of the patients were 40.08 ± 9.38 years, ranging from 20 to 62 years. The majority of study participants were within the WHO stage III category at the baseline. The most widely used HAART regimen in this study was 1e (TDF-3TC-EFV) (Table 1).

**Table 1:** Socio-demographic and clinical characteristics of HIV positive patients taking HAART at Black Lion Specialized Hospital, Addis Ababa, Ethiopia, 2017

Variables	Frequency (*n* = 176)	Percentage (%)
Age (in years)		
20–29	28	15.9
30–39	58	33
40–49	61	34.7
50–59	26	14.8
60–69	3	1.7
Sex		
Male	69	39.2
Female	107	60.8
Marital Status		
Single	50	28.4
Divorced	24	13.6
Married	75	42.6
Widowed	27	15.3
Educational status		
illiterate	21	11.9
Primary school	74	42
High school	62	35.2
Certificate and above	19	10.8
WHO clinical stages at the baseline		
Stage I	32	18.2
Stage II	39	22.2
Stage III	59	33.5
Stage IV	46	26.1
Types of ART regimens		
1c	30	17
1d	40	22.7
1e	94	53.4
1f	12	6.8

Note: 1c = AZT-3TC-NVP, 1d = AZT-3TC-EFV, 1e = TDF-3TC-EFV, 1f = TDF-3TC-NVP

### Platelets and CD4^+^ T cell counts of study participants

The mean platelet count of the study participants were 218.44 ± 106.6 × 10^3^/μl at the baseline and 273.65 ± 83.8 × 10^3^/μl after HAART initiation (*p* < 0.001). Similarly, the mean CD4+ T cell counts showed an increment from 162.35 ± 113.2 cells/μl at the baseline to 360.76 ± 196.2 cells/μl after HAART initiation (*p* < 0.001).

### Prevalence of thrombocytopenia and associated factors before HAART initiation

**Fig. 1:**
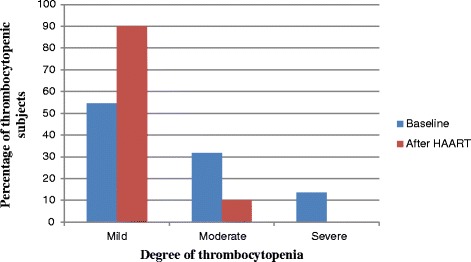
Degree of thrombocytopenia among HIV positive adult patients at baseline and after six months of HAART initiation at Black Lion Specialized Hospital, Addis Ababa, Ethiopia, 2017

The prevalence of thrombocytopenia was 25% before HAART initiation. From the total thrombocytopenic subjects at the baseline, 54.6, 31.8 and 13.6% had mild, moderate and severe thrombocytopenia, respectively (Fig. 1). The overall prevalence of thrombocytopenia was 26.1% among males and 24.3% among females. However, the difference was not statistically significant. In this study, the majority of thrombocytopenia cases (31%) were observed in the aged subjects (≥ 50 years). HIV patients whose CD4 counts less than 200 cells /mm^3^ were 4.4 times more likely to have thrombocytopenia than HIV patients whose CD4 counts greater than or equals to 350cells/mm^3^ (Table 2).

**Table 2:** Thrombocytopenia and its associated factors before HAART initiation in HIV positive patients attending Black Lion Specialized Hospital, Addis Ababa, Ethiopia, 2017

Variables	Thrombocytopenia		Adjusted OR(95%CI)	*P* value
	Yes (%)	No (%)		
Age(in years)				
20–29	3(10.7%)	25 (89.3%)	0.3(0.07–1.24)	
30–39	14(24.1%)	44(75.9%)	0.7(0.26–1.97)	0.45
40–49	18(29.5%)	43(70.5%)	0.9(0.34–2.40)	
≥ 50	9(31%)	20 (69%)	1	
Sex				
Female	26(24.3%)	81(75.7%)	0.9(0.47–1.99)	0.93
Male	18(26.1%)	51(73.9%)	1	
WHO clinical stage				
Stage III/IV	27(25.7%)	78(74.3%)	1.1(0.54–2.34)	0.76
Stage I/II	17(23.9%)	54(76.1%)	1	
CD4 count (cells/mm^3^)				
< 200	34(28.9%)	88(72.1%)	4.4(0.52–36.28)	0.35
200–349	9(22.5%)	31(77.5%)	3.3(0.37–29.84)	
≥ 350	1(7.1%)	13(92.9%)	1	

### Prevalence of thrombocytopenia and associated factors after HAART initiation

The prevalence of thrombocytopenia was 5.7% after HAART initiation, of which 90% had mild and 10% had moderate thrombocytopenia (fig. 1). The prevalence of thrombocytopenia after HAART initiation was significantly decreased by 19.3% (*P* < 0.001). From thrombocytopenic patients after HAART initiation, about 10.1% were males and 2.8% were females. The prevalence of thrombocytopenia was higher (10.3%) among patients whose age group was ≥50 years. HIV patients on AZT based therapy were more likely to have thrombocytopenia than HIV patients on TDF based therapy. Increased percentage of thrombocytopenia were observed in HIV patients whose CD4 count was < 200 cells/μl (*P* < 0.05), but there was no significant association in the thrombocytopenia between patients who were categorized in to different CD4 count categories (*P* > 0.05) (Table 3).

**Table 3:** Thrombocytopenia and its associated factors after HAART initiation in HIV positive patients attending Black Lion Specialized Hospital, Addis Ababa, Ethiopia, 2017

Variables	Thrombocytopenia		Adjusted OR(95%CI)	*P* value
	Yes (%)	No (%)		
Age(in years)				
20–29	2(7.1%)	26 (92.6%)	0.95(0.13–6.98)	
30–39	1(1.7%)	57(98.3%)	0.18(0.02–1.94)	0.53
40–49	4(6.6%)	57(93.4%)	0.66(0.13–3.41)	
≥ 50	3(10.3%)	26 (89.7%)	1	
Sex				
Male	7(10.1%)	62(89.8%)	3.45(0.79–15.05)	0.09
Female	3(2.8%)	104(97.2%)	1	
Types of ART regimen				
TDF based	6(4.8%)	119(95.2%)	0.86(0.22–3.38)	0.83
AZT based	4(7.8%)	47(92.2%)	1	
CD4 count (cells/mm^3^)				
< 200	5(12.2%)	36(87.8%)	3.39(0.74–15.63)	0.19
200–349	2(3.8%)	51(96.2%)	0.96(0.15–6.29)	
≥ 350	3(3.6%)	79(96.3%)	1	

## Discussion

It’s well documented that hematological abnormalities are common in HIV infected patients [2]. Thrombocytopenia, for instance, is a condition frequently seen in HIV infected individuals regardless of HIV status, gender, or age. Consequently, it’s tempting to deduce that the presence of thrombocytopenia is associated with rapid disease progression, and by complicating the management of AIDS patients, thrombocytopenia has become a medical challenge [13].

This study revealed that the prevalence of thrombocytopenia was 25% at baseline and 5.7% after six months of HAART initiation. A study conducted in Uganda reported that the prevalence of thrombocytopenia was 17.8% among antiretroviral HAART-naive and was 13.0% for clients who were on ART for up to 6 months [10]. Another study conducted in Ethiopia reported that the prevalence of thrombocytopenia was 4.1% in patients on HAART and 9% in HAART naive patients [11]. The difference in results seen from the present study might be due to the difference in the definition of thrombocytopenia, study design and size of the study population.

The decrease in the prevalence of thrombocytopenia after HAART initiation might be due to; disorders of hematopoiesis, opportunistic infections and immune causes related to HIV leading to low platelets count could be reverted after HAART initiation [14]. Additionally the presence of intervention protocol for HIV subjects will decrease the incidence of thrombocytopenia [13]**.**

The study found that older age (age > 50 years) was a risk factor for thrombocytopenia, In Lai et al.’s study, for every 1-year increase in age, the prevalence of thrombocytopenia increased by 1.04 fold [15]. The increase in the prevalence of thrombocytopenia with age might be due to a higher incidence of myelodysplasia in older patients [15]. However, thrombocytopenia had not showed statistical significance difference with sex and age. This was in agreement with previous studies [9, 16].

According to the present study, the prevalence of thrombocytopenia was increased with decreasing CD4 count both before and after HARRT initiation. Thrombocytopenia was more prevalent among HIV positive patients who had a CD4 + T cell count of < 200 cells/μl. This finding was consistent with several studies, which reported that thrombocytopenia was more prevalent among patients with CD4 count < 200 cells/mm^3^ [11, 17, 18].

However, the increase in prevalence of thrombocytopenia with decreased CD4 cell count was not statistically significant. This might be due to increase in the frequency of bone marrow abnormalities as the disease progresses [19] and thrombocytopenia is greater in advanced HIV infection [20].

The present study showed that patients on AZT based HAART regimen had a higher prevalence of thrombocytopenia compared to TDF based HAART regimen. Similar to the current finding study done by Suma et al. showed an increment in the prevalence of thrombocytopenia after initiation of Zidovudine [8]. However, thrombocytopenia had not showed statistical significance difference with the type of HAART regimen. The high prevalence of thrombocytopenia might be due to immune mediated destruction of both platelets and megakaryocytes occurs in Zidovudine therapy [8].

## Conclusions

In conclusion, this study has shown that the prevalence of thrombocytopenia after HAART initiation was decreased significantly. HIV patients with old age (age greater than 50 years), lower CD4 + T cell count and AZT based HAART regimen had an increased risk of developing thrombocytopenia. Based on our results, a number of study participants still had thrombocytopenia after initiation of HAART. Therefore continuous screening for thrombocytopenia among HIV infected patients should be performed to decrease the risk of morbidity and mortality.

## Additional file

Additional file 1: Questionnaires The data within additional file 1 contains questionnaires, which were used to collect information from the study participants for this study. The questionnaires had two parts; the first part is for collecting data about socio-demographic characteristics of the study subjects. The second part is for collecting data concerning clinical characteristics and immunohematological profiles of the study participants before and after HAART initiation. (DOCX 19 kb)

## Abbreviations

3TC: Lamivudin
AIDS: Acquired immunodeficiency syndrome
ART: Antiretroviral treatment
AZT/ZDV: Azidothymidine/ Zidovudine
CD4: Cluster of differentiation 4
EVF: Efavirenz
HAART: Highly active antiretroviral therapy
HIV: Human immunodeficiency virus
NVP: Nevirapine
TDF: Tenofovir
