# Supplementary material for: Prevalence of thrombocytopenia before and after initiation of HAART among HIV infected patients at black lion specialized hospital, Addis Ababa, Ethiopia: a cross sectional study
Source: BMC Hematol. 2018 May 9;18:9. doi: 10.1186/s12878-018-0103-6 (PMC5944097; doi:10.1186/s12878-018-0103-6)
Supplement: Supplementary file 1 — Questionnaires The data within additional file 1 contains questionnaires, which were used to collect information from the study participants for this study. The questionnaires had two parts; the first part is for collecting data about socio-demographic characteristics of the study subjects. The second part is for collecting data concerning clinical characteristics and immunohematological profiles of the study participants before and after HAART initiation. (DOCX 19 kb) [file 12878_2018_103_MOESM1_ESM.docx]

## Questionnaires

01 Questionnaire identification No: __________

02 Date of data collection: ____/____/_____

03 Signature of the data collector: _________

04 Checked by supervisor:

Signature_____________ Date ___/___/____

| **Part 1: Socio-demographic characteristics** | | | | | |
| --- | --- | --- | --- | --- | --- |
| No | | Questions | Responses | Skip to | Code |
| 101 | | Age | ________(in years) |  |  |
| 102 | | Sex | 1. Male  2. Female |  |  |
| 103 | | Marital status | 1. Single  2. Divorced  3. Married  4. Widowed |  |  |
| 104 | | Educational status | 1. Illiterate  2. Primary school  3. High school  4. Certificate and above |  |  |
| **Part 2: Clinical characteristics and immuneohematological profiles of the study subjects**  **A: Clinical characteristics of the study subjects** | | | | | |
| 201 | WHO clinical stages of HIV disease at the baseline | | 1.Satge I  2.Stage II  3. Stage III  4. Stage IV |  |  |
| 202 | Type of ART regimens taken by the study subjects | | 1.1C (*AZT-3TC-NVP)*  2. 1d *(AZT-3TC-EFV)*  3. 1e *(TDF-3TC-EFV)*  4. 1f *(TDF-3TC-NVP)* |  |  |
| **B. Immunohematological profiles of the study subjects** | | | | | |
| 203 | CD4 counts at baseline | |  |  |  |
| 204 | CD4 counts after six months of ART initiation | |  |  |  |
| 205 | Platelet counts at baseline | |  |  |  |
| 206 | Platelet counts after six months of ART initiation | |  |  |  |
